# Development and Multi-Data Set Verification of an RNA Binding Protein Signature for Prognosis Prediction in Glioma

**DOI:** 10.3389/fmed.2021.637803

**Published:** 2021-02-09

**Authors:** Chunpeng Sheng, Zhihua Chen, Jianwei Lei, Jianming Zhu, Shuxin Song

**Affiliations:** Department of Neurosurgery, The Second Affiliated Hospital of Nanchang University, Nanchang, China

**Keywords:** RNA binding proteins, signature, glioma, prognosis, single cell RNA sequencing

## Abstract

**Objective:** Increasing evidence emphasizes the clinical implications of RNA binding proteins (RBPs) in cancers. This study aimed to develop a RBP signature for predicting prognosis in glioma.

**Methods:** Two glioma datasets as training (*n* = 693) and validation (*n* = 325) sets were retrieved from the CGGA database. In the training set, univariate Cox regression analysis was conducted to screen prognosis-related RBPs based on differentially expressed RBPs between WHO grade II and IV. A ten-RBP signature was then established. The predictive efficacy was evaluated by ROCs. The applicability was verified in the validation set. The pathways involving the risk scores were analyzed by ssGSEA. scRNA-seq was utilized for evaluating their expression in different glioma cell types. Moreover, their expression was externally validated between glioma and control samples.

**Results:** Based on 39 prognosis-related RBPs, a ten RBP signature was constructed. High risk score distinctly indicated a poorer prognosis than low risk score. AUCs were separately 0.838 and 0.822 in the training and validation sets, suggesting its well performance for prognosis prediction. Following adjustment of other clinicopathological characteristics, the signature was an independent risk factor. Various cancer-related pathways were significantly activated in samples with high risk score. The scRNA-seq identified that risk RBPs were mainly expressed in glioma malignant cells. Their high expression was also found in glioma than control samples.

**Conclusion:** This study developed a novel RBP signature for robustly predicting prognosis of glioma following multi-data set verification. These RBPs may affect the progression of glioma.

## Introduction

Glioma is the most frequently diagnosed primary brain malignancy, accounting for 70% of all brain malignancies (1). In line with the 2016 World Health Organization (WHO) classification, glioblastoma (GBM) is the most common histology type, which corresponds to WHO grade IV, with a median survival of <2 years and a 5-year survival rate of 5% (2). The incidence of lower grade gliomas (LGG) WHO grade II is relatively lower and patients with grade II exhibit better clinical outcomes and more sensitive to therapies (3). At present, the diagnosis of glioma primarily depends on histopathology, imaging as well as molecular diagnosis (4–6). Nevertheless, because of complicacy and heterogeneity, traditionally diagnostic and therapeutic techniques exert side effects for the clinical outcomes of patients. To prolong the survival time of patients, it is of clinical importance for discovering novel accurate molecular biomarkers for prognosis prediction in glioma.

RBPs play major participants of the life cycle of mRNAs (7). One thousand five hundred forty-two RBP genes have been discovered in the human genome, corresponding to over 7.5% of protein-encoding genes (8). Totally, ~50% of RBPs exert post-transcriptional mediated effects on gene expression (8). Abnormally expressed RBPs have been considered as drivers for cancers. They may be involved in regulating the progression and spread of cancers (9). Increasing evidence highlights the critical roles of RBPs in the malignant biological behaviors in gliomas (10). For example, RBP SRSF1 could regulate the cell cycle through stabilizing NEAT1 for glioma (11). RBP PCBP2 may modulate glioma growth via regulation of FHL3 (12). RBP SRSF3 regulates RNA alternative splicing, thereby inducing glioblastoma occurrence via influencing key biological processes (13). Nevertheless, their roles still require to be explored in depth via further functional studies. Recent studies suggest that several RBPs exhibit significant associations with clinical outcomes of glioma patients, such as SNRPN and IGF2BP3 (14). Hence, targeting RBPs appears to be promising strategies for the development of novel treatment against cancers. Moreover, it is of significance to understand the prognostic implications of RBPs in glioma. Herein, our findings developed a RBP signature for predicting the clinical outcomes of glioma patients. After validation, this signature exhibited a robust predictive efficacy.

## Materials and Methods

### Glioma Data Acquirement and Preprocessing

Two glioma datasets (*n* = 693 or 325) containing mRNA sequencing and corresponding clinical information were retrieved from the Chinese Glioma Genome Atlas (CGGA). A dataset (*n* = 693) was used as the training set and another (*n* = 325) was utilized as the validation set. The batch effects were presented after integration of the two datasets.

### Differential Expression Analysis

The mRNA expression profiles of RBPs were extracted from the training and validation sets. Differentially expressed RBPs were defined by comparing WHO grade II and IV samples via the edgeR package in R (15). The criteria were set as follows: |log fold change (FC)| ≥ 0.58 and false discovery rate (FDR) > 0.5. Differentially expressed RBPs were visualized into volcano and heatmap plots.

### Univariate and Multivariate Cox Proportional Hazard Model

Univariate cox regression analysis was conducted for differentially expressed RBPs in the training set. RBPs with hazard ratio > 1 and *p* < 0.001 were risk factors and those with hazard ratio <1 and *p* < 0.001 were protective factors. RBPs with *p* < 0.001 were chosen for multivariable cox regression. Totally, ten RBPs were filtered out for building up the predictive model. The risk score for each sample was calculated by combining the coefficient and the expression level of RBP. Afterwards, the patients were separated into high- and low-risk groups in line with the median values of the risk scores. Kaplan-Meier curves were constructed and the differences in survival time between groups were compared by log-rank test. Using the heatmap package in R, the ten RBPs were displayed between the two groups. Relative operating characteristic curves (ROCs) were built for assessment of the predictive efficacy of the signature. Univariate and multivariate cox regression analysis was utilized to evaluate the associations between risk score as well as other clinical features and prognosis among glioma samples.

### Functional Enrichment Analysis

Kyoto Encyclopedia of Genes and Genomes (KEGG) and Gene Ontology (GO) enrichment analyses were conducted for prognosis-related RBPs via the clusterProfiler package in R (16). Terms with adjusted *p* < 0.05 were statistically significant.

### Single Sample Gene Set Enrichment Analysis (ssGSEA)

Hallmark gene sets were obtained from the Molecular Signatures Database v7.2. ssGSEA was utilized to analyze the associations between risk scores and signaling pathways via the Gene Set Variation Analysis (GSVA) in R package (17). GSVA score in each pathway was calculated for a specific sample. The difference in a specific alteration in a pathway was compared between high and low risk groups via Wilcoxon rank-sum test.

### Construction and Evaluation of a Nomogram

Based on the coefficients of the ten RBPs from the multivariate Cox regression analysis, the score was determined for each RBP. The total point was obtained by adding all scores of the RBPs. Through the function conversion relationship between the total point and the 1-, 2-, and 3-year survival probability, the predicted probability of the individual outcome event was calculated. The nomogram was conducted via the forestplot package in R. Calibration curve was drawn for internal verification utilizing the rms package. The predicted 5-year survival was compared with the actual survival time.

### Single Cell RNA-Sequencing (scRNA-seq) Data

Three single cell RNA-seq datasets for glioma including GSE131928 Smart-seq2 (18), GSE131928 10X Genomics, GSE102130 datasets were retrieved from the Gene Expression Omnibus (GEO; https://www.ncbi.nlm.nih.gov/gds/). Following quality control, normalization and linear scaling analyses, cell cluster was presented via the Seurat package in R (19). Cell types were visualized by the t-distributed stochastic neighbor embedding (t-SNE). The expression levels of the ten RBPs were visualized in each cell type.

### Spearson Correlation Analysis

The expression levels of IGF2BP3, RDM1, NSUN7, EXO1, APOBEC3F, FBXO17, FAM46A, ANG, ADARB2, and EIF4E1B were compared between glioma WHO grade II and IV samples. At the mRNA levels, Spearson correlation analysis was presented between these RBPs among the whole datasets.

### Gene Expression Profiling Interactive Analysis (GEPIA)

Using the GEPIA database; (http://gepia2.cancer-pku.cn/#index), the expression of RBPs was compared between tumor (*n* = 163) and normal (*n* = 207) specimens in the GBM dataset from TCGA and GTE_X_ database. The cutoffs were set as |log2FC| > 1 and *p* < 0.01.

### Reverse Transcription Quantitative Polymerase Chain Reaction (RT-qPCR)

Ten pairs of glioma and normal tissue specimens were collected from The Second Affiliated Hospital of Nanchang university. All patients provided written informed content. This study was approved by the Ethics Committee of The Second Affiliated Hospital of Nanchang university (2020018). Total RNA was extracted from tissues utilizing TRIzol (Beyotime, Shanghai), which was reverse transcribed into cDNA. RT-qPCR was presented by the miScript SYBR Green PCR Kit (Applied Biosystems, USA). Table 1 listed the sequence information of primers. GAPDH was utilized as a control. The expression levels were calculated with the 2–ΔΔCt method. Using GraphPad Prism 7.0, the differences between tumor and normal groups were determined using student's *t*-test. *P* < 0.05 indicated statistically significant.

**Table 1:** The primer sequences for RT-qPCR.

**Gene name**	**Primer sequence (5^**′**^-3^**′**^)**
ANG	CTGGGCGTTTTGTTGTTGGTC (forward) GGTTTGGCATCATAGTGCTGG (reverse)
EXO1	TGAGGAAGTATAAAGGGCAGGT (forward) AGTTTTTCAGCACAAGCAATAGC (reverse)
FBXO17	CTGACCCGGTCCTTCAGTG (forward) CTCCCGTACTGCTCAAAAGATAC (reverse)
IGF2BP3	TATATCGGAAACCTCAGCGAGA (forward) GGACCGAGTGCTCAACTTCT (reverse)
NSUN7	GGACTCCGTTTATGTCATGGC (forward) CTCAGACTCGGACAAGGACC (reverse)

## Results

### Screening Prognosis-Related RBPs for Glioma

**Figure 1:**
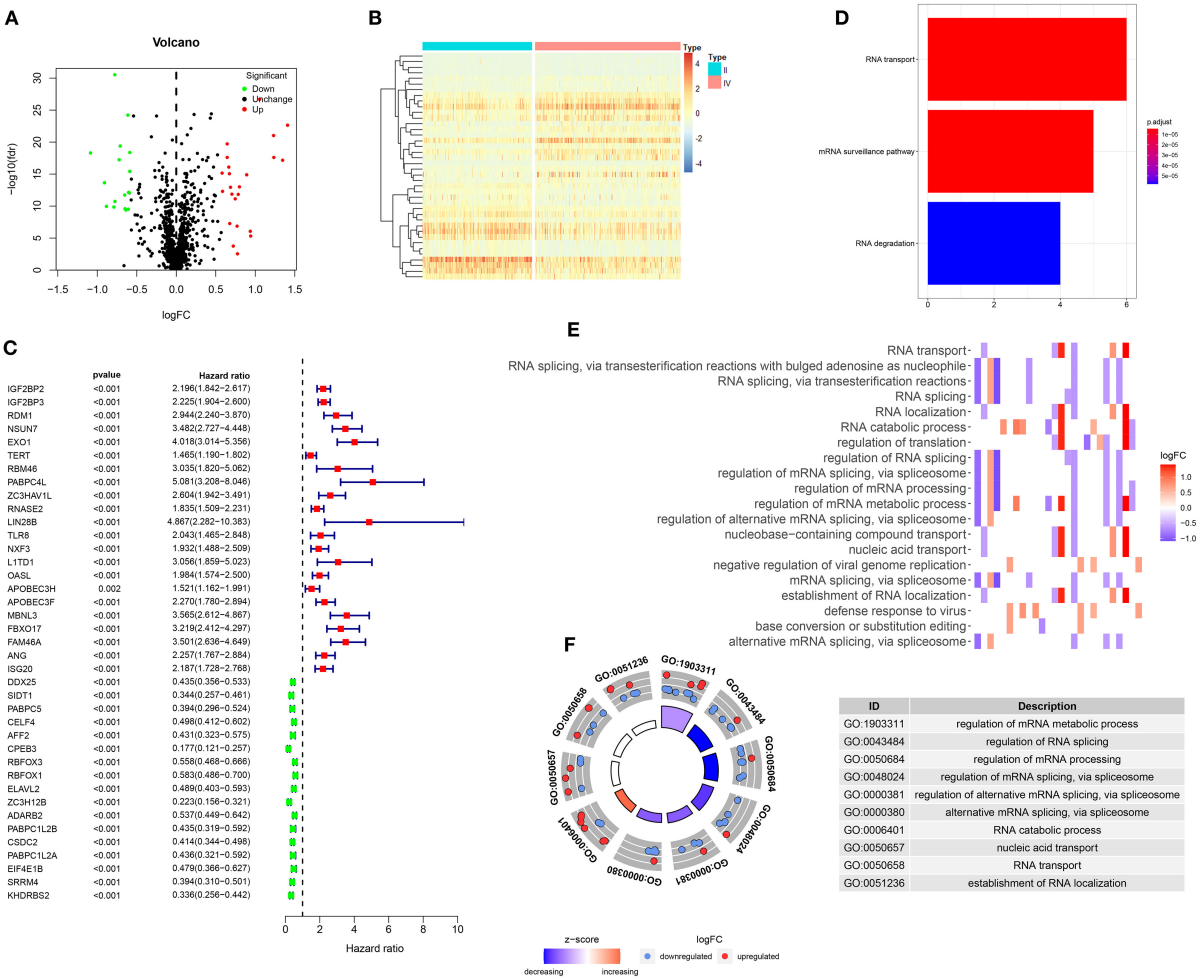
Screening prognosis-related RBPs for glioma. **(A)** Volcano plots for up-regulated (red) and down-regulated (green) RBPs in glioma WHO grade IV compared to grade II samples. **(B)** Hierarchical clustering analysis for differentially expressed RBPs between glioma WHO II and IV samples. **(C)** Univariate cox regression analysis for screening 39 glioma prognosis-related RBPs. **(D)** The signal pathways involved in these RBPs. **(E,F)** Key biological processes enriched by these RBPs.

In this study, we compared the differences in expression of 1,487 RBPs between glioma WHO grade II and IV samples in the training set. With the criteria of |log FC| ≥ 0.58 and FDR > 0.5, 40 RBPs were abnormally expressed between glioma II and IV samples (Supplementary Table 1). Among them, 23 RBPs were up-regulated whereas 17 RBPs were down-regulated in glioma grade IV compared to II (Figure 1A). Heat maps visualized the expression patterns of these RBPs between glioma II and IV samples (Figure 1B). We explored prognosis-related RBPs in glioma via univariate cox regression analysis. Our data suggested that 39 RBPs were significantly associated with prognosis of glioma (Figure 1C). Among them, 22 RBPs were risk factors for glioma and 17 RBPs were protective factors for glioma. To further probe the biological functions of these prognosis-related RBPs, we carried out KEGG and GO enrichment analysis. Our data suggested that RNA transport, mRNA surveillance and RNA degradation pathways were distinctly enriched, as shown in Figure 1D. GO enrichment analysis results revealed that these RBPs were involved in mediating various key biological processes such as mRNA metabolic process, RNA splicing, mRNA processing, RNA transport and localization (Figures 1E,F). These data demonstrated that the prognosis-related RBPs we selected could be involved in the progression of glioma.

### Establishment of an RBP Signature for Prognosis Prediction in Glioma

**Figure 2:**
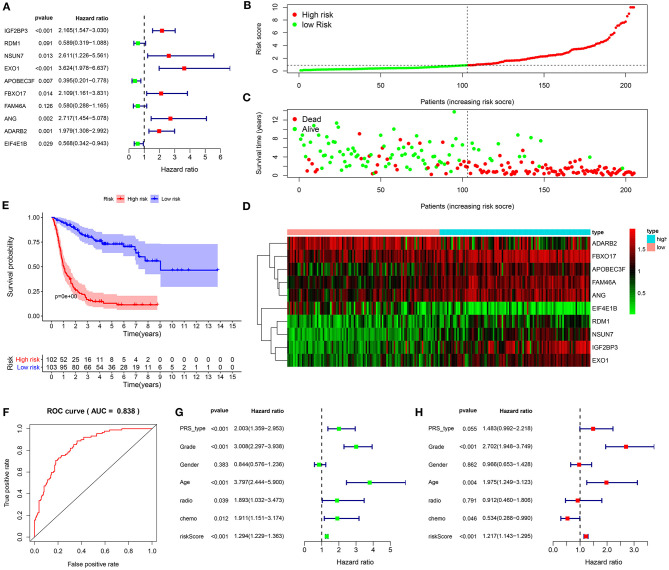
Establishment of a ten-RBP signature for predicting the prognosis of glioma in the training set. **(A)** Multivariate cox regression analysis for the predictive values of the ten RBPs in glioma. **(B)** The ranking of the risk scores among glioma patients. **(C)** Survival status in high and low risk groups. **(D)** Heat map for the expression levels of the RBPs between high and low risk groups. **(E)** Kaplan-Meier curves for high and low risk glioma patients. **(F)** Construction of a ROC curve for assessment of the predictive efficacy of the signature. **(G)** Univariate and **(H)** multivariate cox regression analysis for the association between risk score as well as clinical features and prognosis in glioma.

Based on prognosis-related RBPs, in the training set, a ten-RBP signature was established for glioma via multivariate cox regression analysis (Figure 2A). The risk score for each glioma was determined as follows: 0.772546974482237 ^*^ the expression levels of IGF2BP3 + (-0.529440079138294) ^*^ the expression levels of RDM1 + 0.959791181375875 ^*^ the expression levels of NSUN7 + 1.28748170838841 ^*^ the expression levels of EXO1 + (−0.92777652353892) ^*^ the expression levels of APOBEC3F + 0.746260894529048 ^*^ the expression levels of FBXO17 + (−0.545584467299167) ^*^ FAM46A + 0.999495382483528 ^*^ the expression levels of ANG + 0.682420092015883 ^*^ the expression levels of ADARB2 + (−0.565975323034877) ^*^ the expression levels of EIF4E1B. Among them, IGF2BP3 (HR: 2.165, 95%CI: 1.547–3.030, *p* < 0.001), NSUN7 (HR: 2.611, 95%CI: 1.226–5.561, *p* = 0.013), EXO1 (HR: 3.624, 95%CI: 1.978–3.030, *p* < 0.001), FBXO17 (HR: 2.109, 95%CI: 1.161–3.831, *p* = 0.014), ANG (HR: 2.717, 95%CI: 1.454–5.078, *p* = 0.002) and ADARB2 (HR: 1.979, 95%CI: 1.308–2.992, *p* = 0.001) were negatively correlated with survival time for glioma patients. APOBEC3F (HR: 0.395, 95%CI: 0.201–0.778, *p* = 0.007) and EIF4E1B (HR: 0.568 95%CI: 0.342–0.943, *p* = 0.029) were positively associated with clinical outcomes in glioma patients. On the grounds of the median risk score, these patients were separated into high and low risk groups (Figure 2B). In comparison to the low-risk group, the number of dead patients was higher in the high-risk group (Figure 2C). There were distinct differences in expression levels of these ten RBPs between the two subgroups (Figure 2D). The patients in the high-risk group exhibited an unfavorable prognosis compared to those in the low-risk group (*p* < 0.0001; Figure 2E). The area under the curve (AUC) was 0.838, demonstrating that the signature possessed the well performance for prognosis prediction in glioma patients (Figure 2F). Based on the univariate cox regression analysis, PRS type (HR: 2.003, 95%CI: 1.359–2.953, *p* < 0.001), grade (HR: 3.008, 95%CI: 2.297–3.938, *p* < 0.001), age (HR: 3.797, 95%CI: 2.444–5.900, *p* < 0.001), chemotherapy status (HR: 1.911, 95%CI: 1.151–3.174, *p* = 0.012) and risk score (HR: 1.294, 95%CI: 1.229–1.363, *p* < 0.001) were risk factors for glioma (Figure 2G). We further assessed whether the signature could independently predict the clinical outcomes of glioma patients. Our multivariate cox regression analysis revealed the independency of the predictive efficacy of the signature (HR: 1.217, 95%CI: 1.143–1.295, *p* < 0.001; Figure 2H). Grade (HR: 2.702, 95%CI: 1.948–3.749, *p* < 0.001) and age (HR: 1.975, 95%CI: 1.249–3.123, *p* = 0.004) were both independently predictive of the clinical outcomes of glioma patients. Collectively, this signature could be robustly and accurately predictive of the clinical outcomes for glioma patients.

### Validation of the RBP Signature for Predicting Prognosis of Glioma

**Figure 3:**
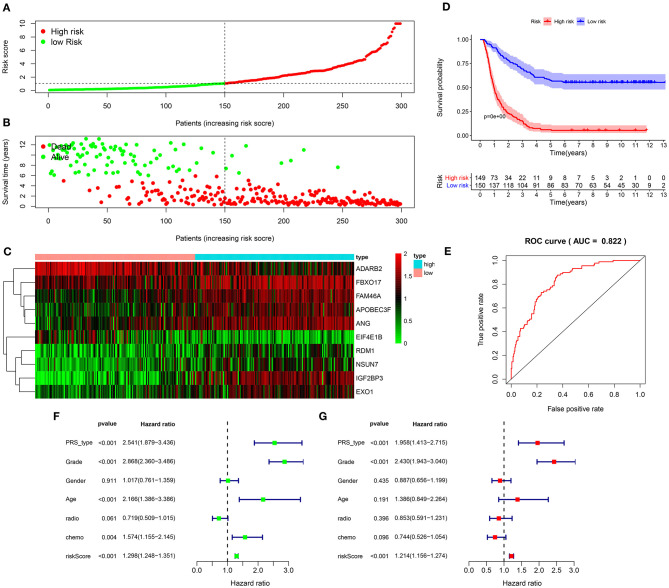
Validation of the RBP signature for predicting prognosis of glioma in the validation set. **(A)** The ranking of the risk scores and **(B)** survival status distribution in high and low risk glioma samples. **(C)** Heat map visualizing the expression levels of the RBPs in high and low risk groups. **(D)** Kaplan-Meier curves for high and low risk patients. **(E)** A ROC curve for evaluating the predictive efficacy of the signature. **(F)** Univariate and **(G)** multivariate cox regression analysis for the associations between risk score as well as clinical characteristics and prognosis in glioma.

We further verified the applicability of the signature for predicting prognosis of glioma in the validation set. Following the same method, patients were divided into high and low risk groups in accordance with the median value (Figure 3A). In the high-risk group, the number of dead patients was much higher than in the low-risk group (Figure 3B). In Figure 3C, these ten RBPs were abnormally expressed between the two subgroups. Patients in the high-risk group exhibited shorter survival time than those in the low-risk group (*p* < 0.0001; Figure 3D). The high predictive value was confirmed by the ROC curve (AUC = 0.822; Figure 3E). PRS type (HR: 2.541, 95%CI: 1.879–3.436, *p* < 0.001), grade (HR: 2.868, 95%CI: 2.360–3.486, *p* < 0.001), age (HR: 2.166, 95%CI: 1.386–3.386, *p* < 0.001), chemotherapy (HR: 1.574, 95%CI: 1.155–2.145, *p* < 0.004), and risk score (HR: 1.298, 95%CI: 1.248–1.351, *p* < 0.001) had negative correlations with prognosis of glioma patients (Figure 3F). Following the multivariate cox regression analysis, risk score (HR: 1.214, 95%CI: 1.156–1.274, *p* < 0.001), PRS type (HR: 1.958, 95%CI: 1.413–2.715, *p* < 0.001), and grade (HR: 2.430, 95%CI: 1.943–3.040, *p* < 0.001) were independent risk factors for glioma (Figure 3G).

### ssGSEA Determines Signaling Pathways Involved in the Ten-RBP Signature

**Figure 4:**
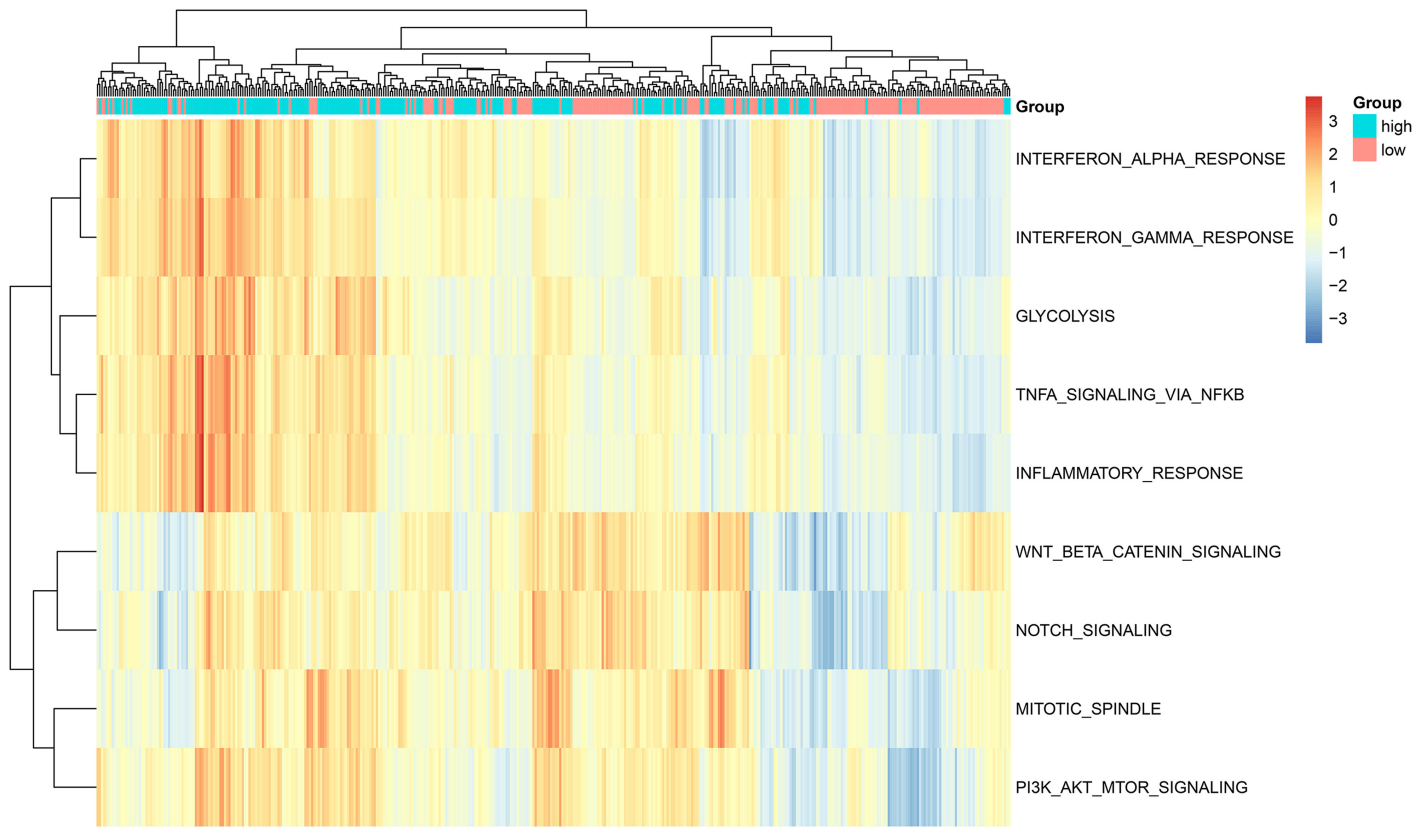
ssGSEA determines the relationships between the ten-RBP signature and signaling pathways for glioma patients.

GSVA score of a specific alteration in a pathway was calculated in each glioma sample. Our data suggested that the high-risk scores were significantly associated with hypoxia, TNFα signaling pathway via NF-κB, inflammatory response, mitotic spindle, PI3K-Akt-mTOR signaling pathway, Notch signaling pathway, interferon α response, interferon γ response and glycolysis (all *p* < 0.0001; Figure 4). No difference in WNT β-Catenin signaling was found between high and low risk score groups. Above findings revealed that cancer-related pathways were activated in the glioma samples with high risk scores.

### Construction and Evaluation of a Nomogram for Prognosis Prediction of Glioma

**Figure 5:**
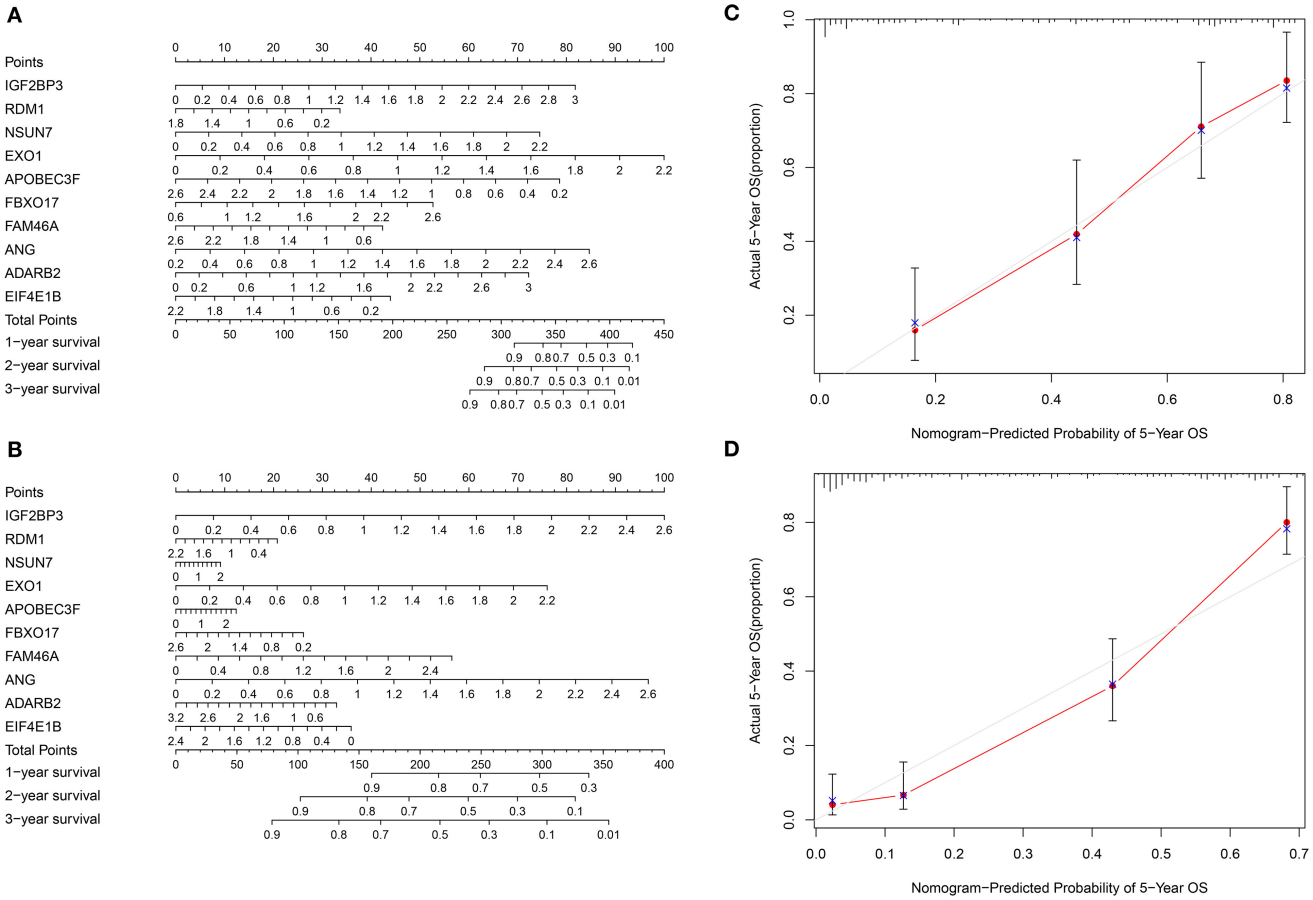
Construction and evaluation of a nomogram for prognosis prediction of glioma. **(A)** A nomogram combining the ten RBPs for predicting the 1-, 2- and 3-year survival time of glioma patients in the training set. **(B)** Evaluation of the nomogram in the validation set. **(C,D)** Calibration curves for assessment of the 5-year survival probability of the nomogram in the **(C)** training and **(D)** validation sets.

To personally calculate the survival rate of specific patient with glioma, a nomogram was established based on the ten RBPs in the training set. According to the influence levels of each RBP in the model on the clinical outcomes, each score of the RBP was assigned, and the total score was determined. The 1-, 2-, and 3-year survival probability of an individual patient was predicted based on the total scores (Figure 5A). We further evaluated the predictive efficacy of the nomogram in the validation set (Figure 5B). Calibration curves were depicted to compare the nomogram-predicted 5-year survival probability and actual survival time. Both in the training (Figure 5C) and validation (Figure 5D) sets, the nomogram exhibited the well performance for prediction of the 5-year survival time for glioma patients. Thus, the nomogram could be used for accurately assessing the survival probability for glioma patients.

### Single Cell RNA-seq Reveals the Expression of the Risk RBP Signatures in Glioma

**Figure 6:**
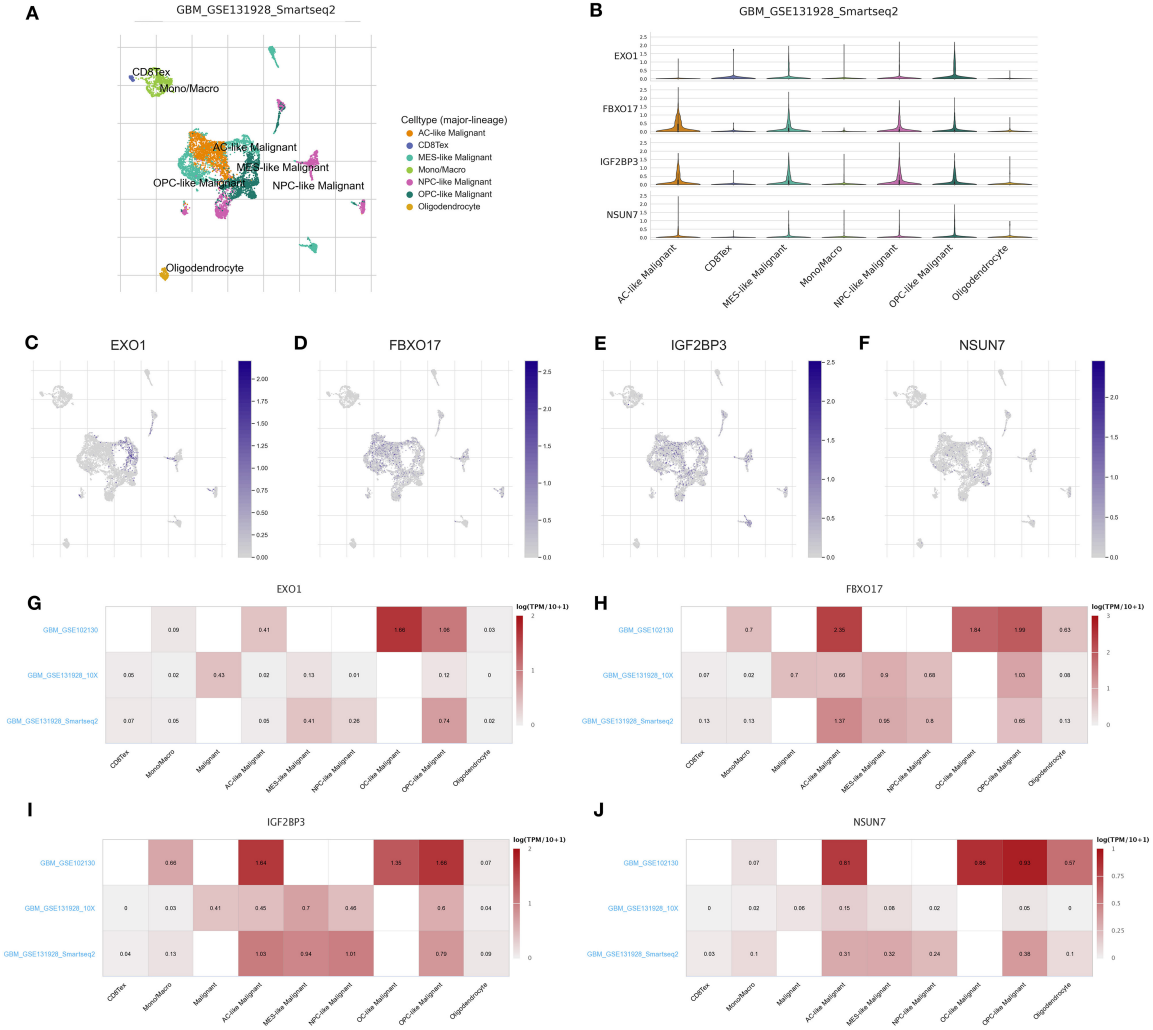
scRNA-seq reveals the expression of the RBP signatures in glioma. **(A)** t-SNE visualizing the seven cell clusters in glioma in the GSE131928 dataset. **(B)** Violin plots for the expression patterns in different cell clusters for IGF2BP3, NSUN7, EXO1, and FBXO17 in the GSE131928 dataset. The expression distributions of **(C)** EXO1, **(D)** FBXO17, **(E)** IGF2BP3, and **(F)** NSUN7 in different cell types in the GSE131928 dataset. Heat maps visualizing the expression patterns of **(G)** EXO1, **(H)** FBXO17, **(I)** IGF2BP3, and **(J)** NSUN7 in different cell types in the GSE131928, GSE131928 and GSE102130 datasets.

We deeply analyzed the expression levels of the risk RBP signatures in a single glioma cell in the three datasets. In the GSE131928 Smart-seq2 dataset, seven cell types were clustered, including AC-like malignant, CD8Tex, MES-like malignant, mon/macrophages, NPC-like malignant, OPC-like malignant and oligodendrocyte (Figure 6A). Consistent with our multivariate Cox regression analysis results, IGF2BP3, NSUN7, EXO1, and FBXO17 were highly expressed in AC-, MES-, NPC-, and OPC-like malignant cells (Figures 6B–F). Furthermore, heat map visualized the expression patterns of EXO1 (Figure 6G), FBXO17 (Figure 6H), IGF2BP3 (Figure 6I), and NSUN7 (Figure 6J) in different cell types in the three datasets. Higher expression levels of the four RBPs were detected in the AC-, MES-, NPC- and OPC-like malignant cells, which could be related to poor prognosis for glioma patients.

### The Risk RBP Signatures Are Highly Expressed in Glioma Grade IV Than II

**Figure 7:**
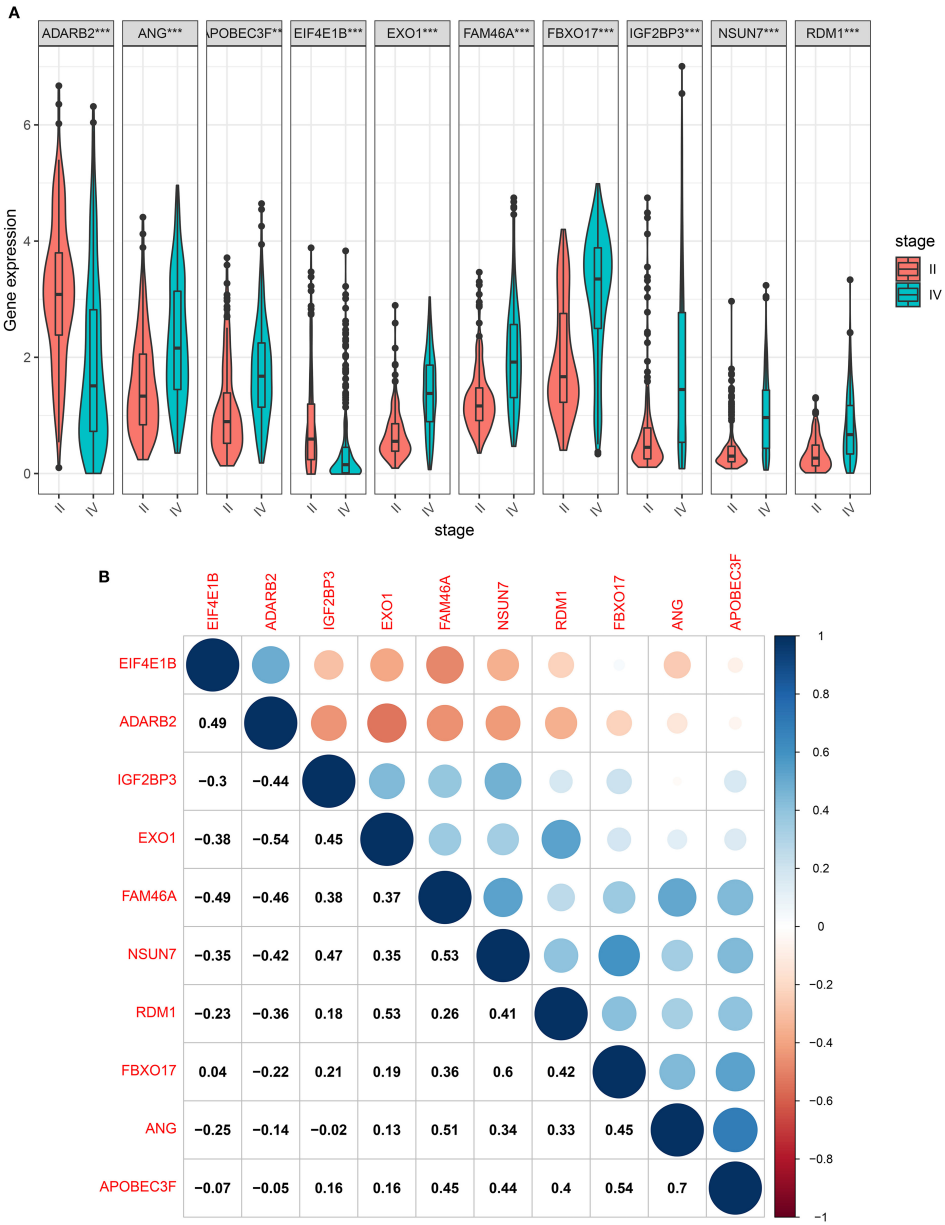
The risk RBP signatures are highly expressed in glioma grade IV than II. **(A)** Box plots showing the expression levels of the ten RBP signatures in glioma grade IV and II samples. ***p < 0.001. **(B)** The correlation between the ten RBP signatures at the mRNA levels. Blue: negative correlation; red: positive correlation. The size of the circle is proportional to the correlation coefficient.

We further compared the expression levels of the ten RBP signatures in glioma grade II and IV samples. The data showed that ADARB2 and EIF4E1B had lower expression levels in glioma grade IV than II samples (both *p* < 0.001; Figure 7A). Most risk signatures exhibited higher expression levels of IGF2BP3, RDM1, NSUN7, EXO1, APOBEC3F, FBXO17, FAM46A, and ANG in grade IV than II. These results demonstrated that these risk signatures could affect the progression of glioma. Our study assessed whether these signatures could interact to promote tumor progression. The correlation analysis showed that, at the mRNA levels, there were obvious positive correlations between most of risk signatures (Figure 7B), such as RDM1 and EXO1 (*r* = 0.53), ANG and FAM46A (*r* = 0.51), APOBEC3F and FBXO17 (*r* = 0.54), APOBEC3F and ANG (*r* = 0.7).

### The Risk RBP Signatures Are Highly Expressed in Glioma Than Controls

**Figure 8:**
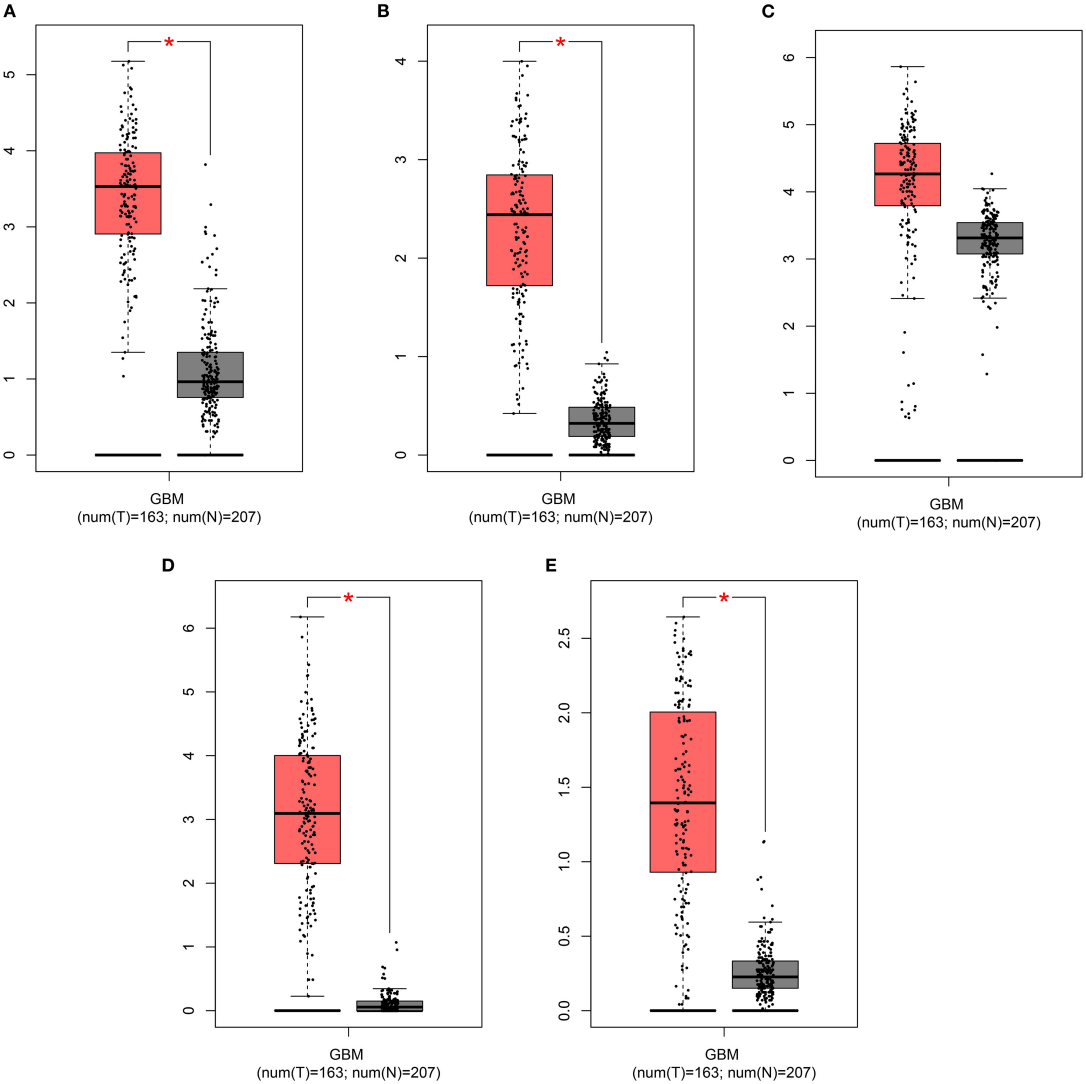
GEPIA displays the highly expressed risk RBP signatures in glioma than controls. Box plots depicting the expression of **(A)** ANG, **(B)** EXO1, **(C)** FBXO17, **(D)** IGF2BP3, and **(E)** NSUN7 in glioma and control samples. *p < 0.01.

**Figure 9:**
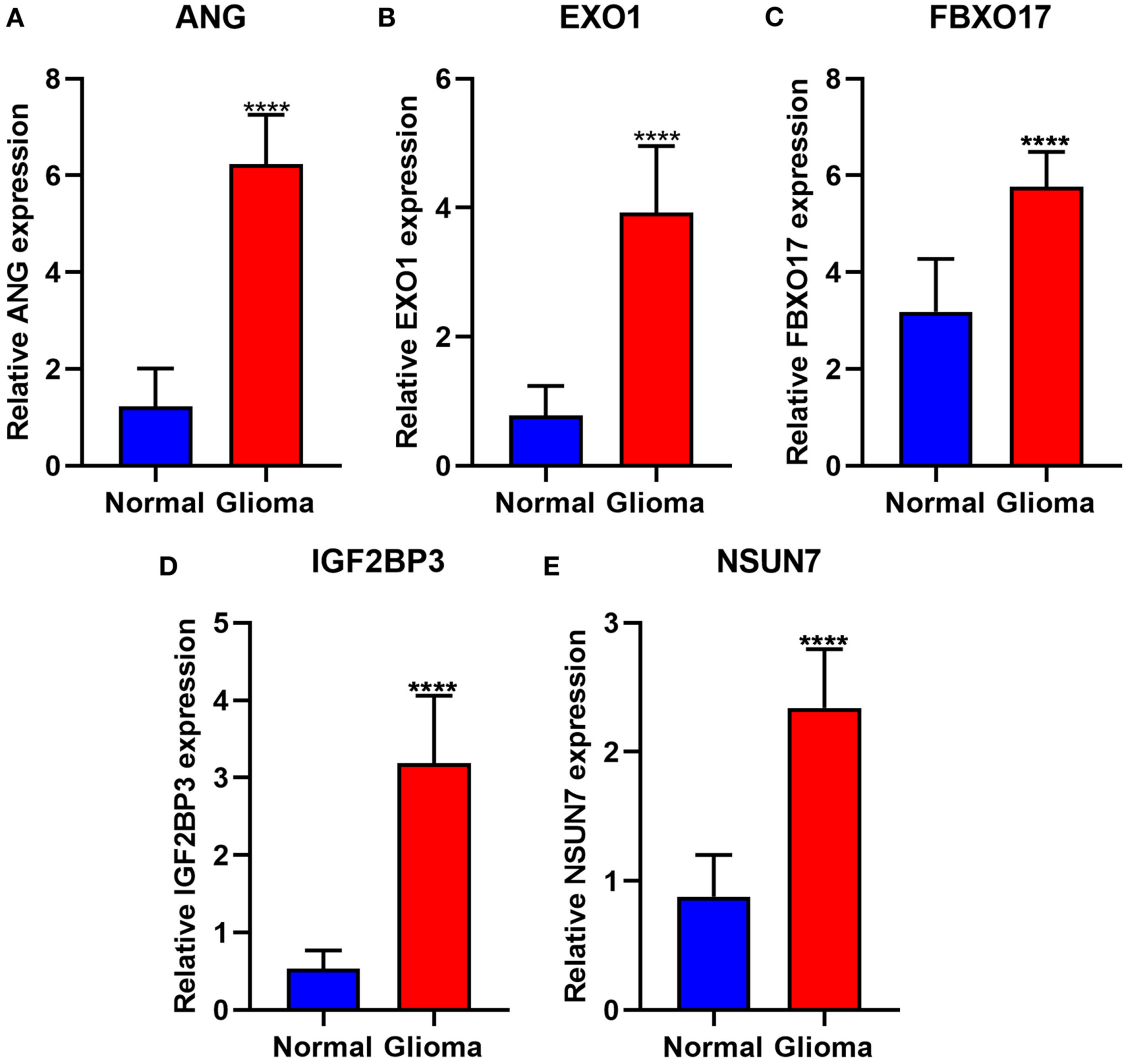
Validation of the highly expressed risk RBP signatures in glioma and normal samples via RT-qPCR. **(A)** ANG, **(B)** EXO1, **(C)** FBXO17, **(D)** IGF2BP3, and **(E)** NSUN7. ****p < 0.0001.

The expression of risk RBP signatures was compared in glioma (*n* = 163) and control samples (*n* = 207) in TCGA-GTEx database. The results showed that ANG (Figure 8A), EXO1 (Figure 8B), FBXO17 (Figure 8C), IGF2BP3 (Figure 8D), and NSUN7 (Figure 8E) displayed distinctly higher expression levels in glioma compared to controls (*p* < 0.05). Furthermore, we validated their expression in 10 pairs of glioma and normal tissue specimens by RT-qPCR. Our data confirmed the up-regulation of ANG (Figure 9A), EXO1 (Figure 9B), FBXO17 (Figure 9C), IGF2BP3 (Figure 9D), and NSUN7 (Figure 9E) in glioma than normal samples (all *p* < 0.0001). These findings revealed that these risk signatures could participate in the progression of glioma.

## Discussion

Recent research demonstrates that several RBPs have closely correlations with the malignant behaviors of glioma (14). Nevertheless, the functions of most of the RBPs in glioma tumorigenesis remain unclear (20). In this study, based on prognosis-related RBPs, we established a ten-RBP signature for prognosis prediction in glioma. Following multi-dataset verification, this signature could robustly predict clinical outcomes for glioma patients.

Herein, 40 RBPs were differentially expressed between glioma WHO grade IV and II. Among them, 39 RBPs were correlated to prognosis of glioma, which might be involved in the malignancies of glioma. Hence, we probed the biological functions involving these RBPs. Our data suggested that these RBPs could post-transcriptionally mediate gene expression via the interaction with targeted RNAs, such as RNA transport, degradation, splicing and localization. Based on them, we developed a ten-RBP signature for glioma. The signature could be utilized for robustly predicting the prognosis of patients. Both in the training and validation sets, high risk score was predictive of poorer prognosis. AUCs were 0.838 and 0.822 in the two sets, respectively, suggesting the well performance. Following adjustment with other clinical features, the signature was an independent prognostic factor. As previous studies, several RBP signatures have been constructed for other cancers. For example, Li et al. built up a nine-RBP gene signature for lung squamous cell carcinoma prognosis (21). A six-RBP model constructed by Wu et al. demonstrated a well performance for predicting the clinical outcomes of bladder cancer (22). However, so far, there is still a lack of RBP-related signature for glioma. Our research fills this gap at some extents.

Our findings revealed that high-risk scores were markedly associated with various cancer-related pathways, like hypoxia, TNFα signaling pathway via NF-κB, inflammatory response, mitotic spindle, PI3K-Akt-mTOR, Notch, interferon α response, interferon γ response and glycolysis pathways, indicating that cancer-related pathways were activated in glioma samples with high-risk scores. These RBPs could affect glioma progression via mediating above pathways. Previously, RBP ZEB1 facilitated hypoxia-mediated epithelial-mesenchymal transition in glioma cells (23). RBP MOV10 that bound to circ-DICER1 induced the proliferation, migration as well as tube formation in glioma cells via activation of PI3K/Akt pathway (24). RBP Musashi1 modulated the proliferative capacities of glioma cells via Notch and PI3K/Akt pathways. Hence, activation of cancer-related pathways could be related to poor prognosis in high-risk score patients. To improve the clinical application potential of RBPs, we constructed a nomogram combining the ten RBPs for assessment of the 1-, 2-, and 3-year survival probability. This nomogram was validated in the validation set, indicating the high application values. After evaluation of calibration curves, the nomogram-predicted 5-year survival was highly consistent with the actual survival, suggesting that the nomogram possessed the potential as a scoring tool for predicting the clinical outcomes of patients.

We further explored why the high-risk scores indicated poor clinical outcomes. ScRNA-seq technology is widely used in basic scientific research and clinical research (25). Single cells have a place in many fields and are of great significance for the early diagnosis, tracking and individualized treatment of cancer (26, 27). The information displayed by traditional sequencing methods is also average information at the multi-cell level, while sequencing at the single-cell level can completely reflect the transcriptome status of different cells in the same cell group (28). Due to the high heterogeneity and complexity of glioma, the survival time of patients in the same pathological stage is completely different. It is of significance to present scRNA-seq analysis for glioma. Herein, we detected the expression of the risk RBP signatures in different glioma cell types. Our data suggested that IGF2BP3, NSUN7, EXO1, and FBXO17 were highly expressed in AC-, MES-, NPC-, and OPC-like malignant cells, which could be related to poor prognosis for glioma patients. Furthermore, the four RBPs exhibited higher expression in WHO grade IV than II, indicating that they were associated with the malignancy of glioma. After validation by RT-qPCR, IGF2BP3, NSUN7, EXO1, and FBXO17 had higher expression levels in glioma than controls. As previous studies, IGF2BP3 facilitated viability and migration for glioma cells (29). NSUN7 is a risk factor for lower-grade glioma patients (30). EXO1 is associated with shorter survival time and hyposensitivity to temozolomide treatment in glioma (31). High FBXO17 expression could independently predict the clinical outcomes for high-grade glioma (32). Combining our results and previous research, these risk RBPs could accelerate the malignant behaviors of glioma.

In this study, a ten-RBP signature was constructed for glioma prognosis. This signature could independently predict the prognosis of patients with high accuracy and sensitivity. Combining RNA-seq and scRNA-seq analysis, we found that the four risk RBPs could contribute to the malignant behaviors of glioma. Their roles should be investigated by basic experiments and larger cohorts.

## Conclusion

The 10-RBP gene signature exhibited a predictive efficacy for glioma prognosis under multi-data set verification. This signature may possess a promising application for clinical decision-making as well as individualized therapy.

## Data Availability Statement

The original contributions presented in the study are included in the article/Supplementary Material, further inquiries can be directed to the corresponding author/s.

## Ethics Statement

The studies involving human participants were reviewed and approved by the Ethics Committee of The Second Affiliated Hospital of Nanchang university (2020018). The patients/participants provided their written informed consent to participate in this study.

## Author Contributions

SS conceived and designed the study. CS and ZC conducted most of the experiments and data analysis, and wrote the manuscript. JL and JZ participated in collecting data and helped to draft the manuscript. All authors contributed to the article and approved the submitted version.

## Conflict of Interest

The authors declare that the research was conducted in the absence of any commercial or financial relationships that could be construed as a potential conflict of interest.

## Supplementary Material

Supplementary Table 1 A list of 40 differentially expressed RBPs between glioma WHO grade II and IV samples.

## Abbreviations

RBPs: RNA binding proteins
WHO: World Health Organization
LGG: lower grade gliomas
CGGA: Chinese Glioma Genome Atlas
FC: fold change
FDR: false discovery rate
ROCs: relative operating characteristic curves
KEGG: Kyoto Encyclopedia of Genes and Genomes
GO: Gene Ontology
ssGSEA: single sample Gene Set Enrichment Analysis
GSVA: Gene Set Variation Analysis
scRNA-seq: single cell RNA-sequencing
GEO: Gene Expression Omnibus
t-SNE: t-distributed stochastic neighbor embedding
GEPIA: Gene Expression Profiling Interactive Analysis
AUC: area under the curve.
